# Clinical Profile of Patients Presenting With Sepsis to the Emergency Department of a Tertiary Care Hospital in Wardha During the COVID Pandemic (June 2020-June 2021)

**DOI:** 10.7759/cureus.29528

**Published:** 2022-09-24

**Authors:** Govind Nagdev, Gajanan Chavan, Gaurav Sahu

**Affiliations:** 1 Department of Emergency Medicine, Jawaharlal Nehru Medical College, Datta Meghe Institute of Medical Sciences, Wardha, IND; 2 Department of Medicine, Jawaharlal Nehru Medical College, Datta Meghe Institute of Medical Sciences, Wardha, IND

**Keywords:** serum lactate, covid 19, sirs criteria, qsofa, sepsis

## Abstract

Background

Sepsis is defined as life-threatening organ dysfunction due to a dysregulated host response to infection. Septic shock, multi-organ dysfunction, and death occur in severe cases with reduced blood flow to vital organs. Sepsis contributes to 15-20% of all global deaths. Through this study, we intend to evaluate the clinical profile and study the common blood investigatory panels along with organisms causing sepsis in patients presenting with sepsis in the emergency department during the COVID pandemic. In addition, the study was also done to estimate the prevalence of sepsis and compare patients having sepsis with serum lactate, sepsis with Systemic Inflammatory Response Syndrome (SIRS) criteria, and sepsis with quick Sepsis Related Organ Failure Assessment (qSOFA) score.

Method

Observational retrospective study to evaluate patients presenting with sepsis diagnosed by the Third International Consensus Definitions for Sepsis and Septic Shock” criteria presenting to the emergency department of Acharya Vinoba Bhave Rural Hospital (AVBRH) affiliated to Jawaharlal Nehru Medical College (JNMC), Wardha during COVID pandemic (June 2020-June 2021).

Results

The majority of the patients presented with fever (42%), and very few presented with altered mental status (8%). Seventy-four percent of the study population did not show any bacterial growth on blood culture, but out of the remaining 26%, blood culture, *Staphylococcus aureus*, *Pseudomonas aeruginosa*, and *Klebsiella pnemoniae* were the significant microbes. Amongst qSOFA, SIRS criteria, and serum lactate as a screening tool for sepsis, SIRS is the most sensitive for screening sepsis patients.

Conclusion

*Staphylococcus aureus, Pseudomonas aerugenosa, and Klebsiella pneumoniae* were the major contributors in the development of sepsis in COVID-19-associated infection. The presence of raised leukocyte counts and serum lactate should alarm clinicians of possible sources of infection. The timely initiation, rapid de-escalation of empirical antibiotics, and strict compliance with infection control practices should be accomplished to reduce the occurrence of multidrug resistance organisms.

## Introduction

Sepsis is defined as life-threatening organ dysfunction due to a dysregulated host response to infection [1]. It's a condition with a wide range of symptoms, including substantial inflammation and organ damage. Due to the wide diversity of clinical presentations, estimates of the annual incidence of severe sepsis range from 300 to 1000 cases per 1 lakh per year [2]. In severe cases, restricted blood flow to key organs leads to septic shock, multi-organ failure, and death.

The most common organisms causing sepsis majorly are gram-positive bacteria [3]. Methicillin-resistant *Staphylococcus Aureus* (MRSA), vancomycin-resistant *Staphylococcus Aureus* (VRSA), and other multidrug-resistant bacteria also contribute to the diverse range of organisms causing sepsis [4]. Furthermore, the fungus is a more common source of infection in immunocompromised patients.

Sepsis is a leading cause of death in hospitals, with fatality rates ranging from 15-20% [5]. It is the most common cause of multi-organ dysfunction syndrome in the world [6]. Post sepsis syndrome is a common condition developed by the patients who survive sepsis, experiencing long-term functional and cognitive deficits, which leads to poor Physical Quality of Life Index (PQLI) and, therefore, loss of productivity [7]. In the United States, sepsis is the tenth leading cause of death among the older age group. Whereas in India, the sepsis cases alone contributed to 11.3 million, with 2.9 million deaths as of reports in 2017 [8]. Sepsis pathogenesis is complicated and multifactorial. Infection-induced pro-inflammatory and anti-inflammatory responses help to prevent tissue damage that leads to organ failure [9]. Endothelial damage causes coagulation diseases such as microvascular thrombi, fibrinolysis, intravascular coagulation, and a decrease in tissue oxygenation. These changes, along with hypotension and vasodilation, lead to organ failure [10].

Septic shock is characterized as sepsis with raised lactate levels >2mmol/l and requiring vasopressors to maintain a mean arterial pressure >65mmhg. Systemic inflammatory response syndrome (SIRS) criteria is a crude means of stratification of patients with systemic inflammation [11]. A respiratory rate of >20/min, a pulse of >90/ min, total leukocyte count (TLC) of <4000 or >12000, a temperature of <36°c or >38 °c, and a score of two or more are considered as significant.

Quick Sequential Organ Failure Assessment (qSOFA) is a method of screening to detect patients who are at a greater risk of mortality. This criterion includes altered mental status, systolic blood pressure of ≤100 mmHg, and respiratory rate of ≥22/min. A score of more than two signifies a high risk of poor outcomes [12, 13].

The present study was done to see any changes in the presentation of sepsis patients during the COVID-19 pandemic by evaluating the clinical profile and studying the investigation panels along with the organisms causing sepsis. In addition, the study was also done to compare risk scores and their accuracy in detecting sepsis.

## Materials and methods

We conducted a retrospective observational study in a tertiary care hospital, Acharya Vinoba Bhave Rural Hospital (AVBRH) affiliated with Jawaharlal Nehru Medical College (JNMC), located in the center of the country in the Vidharbha region, Maharashtra. The study was conducted during the COVID pandemic from June 2020 to June 2021. All patients above 15 years of age who presented to the emergency department (ED) and had clinical features suggestive of sepsis based on qSOFA or raised serum lactate or SIRS criteria were included. We excluded pediatric patients and patients with trauma-induced sepsis. A total of 50 patients were included in the study who fulfilled the inclusion criteria. Cultures were collected depending on the presentation of the patient. Data was collected and evaluated according to SPSS version 16 (IBM Inc., Armonk, New York).

Study procedure

Demographics and Laboratory Tests

Every patient coming to the ED and was eligible according to the study design was separately asked for additional demographic details, which included the age and gender of the patient, along with the signs and symptoms experienced by them, which included fever, heartburn, abdominal pain, any breathing difficulty, and also examined for altered mental status, followed by a physical examination to support the signs and symptoms along with heart rate, respiratory rate, blood pressure, and body temperature. 

Simultaneously blood samples were sent for culture and testing for various parameters, including lactate, WBC, platelet, urea, creatinine, sodium, potassium, alkaline phosphatase (ALP), aspartate aminotransferase (AST), alanine transaminase (ALT), total protein, albumin, and bilirubin. In addition, various other samples, including ascites fluid, pleural fluid, sputum sample, and urine samples, were sent for culture.

Each individual had to undergo mandatory COVID-19 real-time reverse transcriptase-polymerase chain reaction (RT-PCR).

Since this was a retrospective observational study, all the data was collected from the online information system built in the hospital using unique identification numbers (IPD numbers)

Study Tools

Sepsis can be difficult for primary care providers to define, identify early and start treatment. Therefore multiple sepsis scoring tools have been developed, but a gold standard tool does not currently exist. National Early Warning Score (NEWS), Prehospital Early Sepsis Detection (PRESEP) score, Sequential Organ Failure Assessment (SOFA), quick Sequential Organ Failure Assessment (qSOFA), Sepsis Patient Evaluation in the Emergency Department (SPEED), Mortality in Emergency Department Sepsis (MEDS), and SIRS are few of the sepsis scoring criteria.

We used the qSOFA, SIRS, and serum lactate levels to estimate and detect sepsis. qSOFA consists of three attributes: respiratory rate (RR) of ≥22 breaths per minute, altered mentation (Glasgow Coma Scale of <15), and systolic blood pressure (SBP) of <100. A score of two or more points near the onset of infection was associated with a greater risk of death [14].

SIRS has four attributes: heart rate (>90 beats/min), respiratory rate (>20 breaths/min), body temperature (<36°C or >38 °C), and a WBC count (<4000 or >12000). A score of two or more points is considered to be significant.

An elevated serum lactate level of 2 mmol/L or more is associated with mortality in patients with suspected infection and sepsis [15]. Patients with suspected sepsis whose lactate levels are elevated or normal are significantly more likely to be diagnosed with sepsis. The presence of lactate alone, however, cannot rule out the diagnosis on its own due to its lack of sensitivity or specificity [16].

## Results

In age distribution, the maximum number of patients were in the age group between 30-60 years, i.e., 74%, 20% were above 60 years, and 6% were below the 30-year age group. Males were more affected than females, with approximately a 2:1 ratio (Table 1).

**Table 1:** Age and gender distribution of study participants

Factors		Frequency (n=50)	Percent
Age group	Less than 30	3	6.00%
	30 to 60	37	74.00%
	More than 60	10	20.00%
Gender	Female	16	32.00%
	Male	34	68.00%

Amongst 50 patients, the maximum number of patients presented with clinical features of fever (42%) and a few of them with an ulcerated lesion on the foot (10%), abdominal pain (16%), difficulty in breathing (16%), and altered mental status (8%) (Table 2).

**Table 2:** Symptom distribution of study participants

Symptoms		Frequency (n=50)	Percent
Fever	No	29	58.00%
	Yes	21	42.00%
Ulcer	No	45	90.00%
	Yes	5	10.00%
Abdominal pain	No	42	84.00%
	Yes	8	16.00%
Breathing difficulty	No	42	84.00%
	Yes	8	16.00%
Altered mental status	No	46	92%
	Yes	4	8%

Tables 3 and Table 4 show the blood picture and various blood parameters that were performed and were required to make the diagnosis of sepsis according to the different criteria of the risk assessing tools.

**Table 3:** Various parameters RR - respiratory rate; WBC  - white blood cell count; ALP - alkaline phosphatase; AST - aspartate aminotransferase; ALT - alanine transaminase

Factors	Mean	Std. deviation	Std. Error Mean	p-value
Age	49.74	14.389	2.035	< 0.001
Lactate	3.15	1.275	0.180	< 0.001
RR	20.86	3.736	0.528	< 0.001
WBC	18154	9833.288	1390.637	< 0.001
Platelet	1.84	1.146	0.162	< 0.001
Urea	81.52	61.749	8.733	< 0.001
Creatinine	2.74	2.353	0.333	< 0.001
Sodium	140.86	7.600	1.075	< 0.001
Potassium	4.58	0.960	0.136	< 0.001
ALP	145.00	127.338	18.008	< 0.001
AST	260.68	586.349	82.922	0.003
ALT	162.36	380.117	53.757	0.004
Total protein	5.81	1.221	0.173	< 0.001
Albumin	2.70	0.639	0.090	< 0.001
Bilirubin	2.79	5.300	0.750	0.001

**Table 4:** Parameters to suspect sepsis RR - respiratory rate; SBP - systolic blood pressure

Parameter	Range	Frequency (n=50)	Percentage
Total Leucocyte Count	More than 12000	40	80.00%
	Less than 4000	6	12.00%
	4000-1200	4	8.00%
RR	More than 20	32	64.00%
	Less than 20	18	36.00%
SBP	More than 100	16	32.00%
	Less than 100	34	68.00%
Temperature	Less than 97	16	32.00%
	97 to 100	2	4.00%
	More than 100	32	64.00%
Serum lactate	More than 2	38	24.00%
	Less than 2	12	76.00%

Blood culture was done in all 50 patients, amongst which 13 patients showed growth of the organism. Amongst those 13 positive patients, a majority (seven) were of *Staphylococcus aureus*, four patients were *Pseudomonas aeruginosa*-positive, and *Klebsiella pnemoniae* and *Escherichia coli* showed in one patient each. The ascitic fluid culture was done in five patients showing no growth. The pleural fluid culture was done in five patients, of which two were negative, and *Klebsiella pnemoniae*, *Mycobacterium tuberculosis,* and *Acinetobacter *were detected in one patient each. Sputum was done in seven patients, of which two were negative, four were positive for *Klebsiella pnemoniae,* and one was *Mycobacterium tuberculosis-*positive. Fifteen patients underwent urine culture, of which five were negative, Candida was detected in five, *Escherichia coli* in three, and *Enterococcus *and *Pseudomonas *both in one patient each, as shown in Table 5.

**Table 5:** Distribution of organisms causing sepsis by blood and various other culture-wise distribution of study participants

Cultures	Findings	Frequency (n=50)	Percent
Blood culture findings	Escherichia coli	1	2.00%
	Klebsiella pneumoniae	1	2.00%
	Pseudomonas	4	8.00%
	Staphylococcus aureus	7	14.00%
	No growth	37	74.00%
Ascites fluid culture	No growth	5	10.00%
	Not done	45	90.00%
Pleural fluid culture	Acinetobacter specie	1	2.00%
	Klebsiella pneumoniae	1	2.00%
	Mycobacterium tuberculosis	1	2.00%
	No growth	2	4.00%
	Not done	45	90.00%
Sputum culture	Klebsiella pneumoniae	4	8.00%
	Mycobacterium tuberculosis	1	2.00%
	No growth	2	4.00%
	Not done	43	86.00%
Urine culture	Candida specie	5	10.00%
	Escherichia coli	3	6.00%
	Enterococcus	1	2.00%
	Pseudomonas aeruginosa	1	2.00%
	No growth	5	10.00%
	Not done	35	70.00%

Amongst 50 patients with sepsis, 16 patients were COVID-19 positive and 34 tested negative for COVID-19 (Table 6).

**Table 6:** Prevalence of COVID infection in sepsis patients

Prevalence of COVID infection	Frequency (n=50)	Percent
Absent	34	68.00%
Present	16	32.00%

Amongst qSOFA, SIRS criteria, and Serum lactate as a screening tool for sepsis, SIRS is the most sensitive for screening sepsis patients. Patients with sepsis who might benefit from prompt and aggressive treatment were tested qSOFA negative, so it is currently recommended to use full SOFA score or SIRS criteria for risk stratification or predicting disease progression (Table 7).

**Table 7:** Comparison between screening tool of sepsis by serum lactate, SIRS criteria, and qSOFA score SIRS - systemic inflammatory response syndrome; qSOFA - quick Sepsis Related Organ Failure Assessment

Sepsis by various criteria		Frequency (n=50)	Mean ± SD	F test	p-value
qSOFA	Absent	25 (50.0%)	0.44 ± 0.51	186.45	< 0.001
	Present	25 (50.0%)	2.16 ± 0.37		
SIRS	Absent	9 (18.0%)	0.78 ± 0.44	69.750	< 0.001
	Present	41 (82.0%)	2.68 ± 0.65		
Serum lactate	Absent	12 (24.0%)	1.79 ± .14	27.407	< 0.001
	Present	38 (76.0%)	3.57 ± 1.17		

## Discussion

In this study, we analyzed various parameters of 50 sepsis-identified patients admitted to the emergency department of a tertiary care central India hospital during the pandemic in order to see changes in the presentation of sepsis patients during that period and evaluate the performance of SIRS, qSOFA, and serum lactate scores to predict sepsis under this setting. About 68% of our study population were men, which was higher when compared to previous studies [10]. Males were more susceptible to sepsis than females as female sex hormones have a protective effect against infection. Other reason could be the male predominance in India as they are more exposed than women, who typically stay at home [17-19]. Our patients were relatively young, with a mean age of 49.7 years when compared to previous studies from India (56 years), Australia (60.7 years), and Germany (67 years) [20].

Amongst 50 patients with sepsis, 16 tested COVID-19 positive with RT-PCR. COVID-19 causes fever and cough as its primary clinical manifestations, and other features like anorexia, arthralgia, nausea, vomiting, and diarrhea were less prevalent [21]. Since COVID-19 can present in a range of ways, diagnosing it can be difficult because the case definition of sepsis includes fever, yet patients may not always experience fever, and we may end up misdiagnosing the condition. Therefore, we attempted to study the presenting feature in the COVID setting and found that out of 50 patients in our study, 21 (42.0%) of them had a fever which was the chief presentation in comparison to other features.

Umemura et al. conducted a nationwide prospective cohort study in Japan on the spectrum of causative pathogens in sepsis. They included 1184 patients in the study and found out that the most common pathogen was *Escherichia coli* (21.5%), followed by *Klebsiella pneumoniae* (9.0%). Patients with methicillin-resistant *Staphylococcus aureus* had the greatest mortality rate, 47.5%, which was strongly associated with a higher chance of dying [22]. Similar study was done by Legese et al. and found *Klebsiella pneumoniae* (26.1%), *Klebsiella variicola* (18.1%), and *Escherichia coli* (12.4%) as the major causative agent in 1416 blood culture [23]. Our study showed *Staphylococcus aureus *(14.0%) as the major causative organism, followed by *Pseudomonas *(8.0%) and then *Klebsiella pneumoniae* and *Escherichia coli,* both 2.0%, each amongst the blood culture positive plates.

Amongst qSOFA, SIRS, and serum lactate, SIRS criteria was the most sensitive to detect sepsis and predict disease progression. In our study, serum lactate was raised in 76% of patients, and guidelines have mentioned that lactate alone is neither sensitive nor specific enough. Hence it is recommended to use serum lactate as an adjunctive test [24]. This finding was comparable to a prospective cohort study done on patients with clinically confirmed 153 secondary peritonitis patients in a tertiary teaching hospital in Uganda. They concluded that predicting death and a prolonged hospital stay in patients with secondary peritonitis, SIRS is more accurate than qSOFA. Overall, nevertheless, both scores demonstrated poor discrimination in the outcomes, making them ineffective measuring instruments [25].

Studies have shown that qSOFA is more specific but less sensitive than having two of four SIRS criteria for early identification of infection-induced organ dysfunction [26]. Another study conducted on 201 patients concluded that qSOFA had greater predictive capacity as compared to SOFA and SIRS in that order [27].

Limitations

This was a unicenter study with a limited sample size, and only those patients who presented to ED were enrolled. Outcomes of only ED admitted cases were studied. The present study did not follow up with patients after discharge who may have been readmitted or died or need for ventilator, vasopressor, or renal replacement therapy. The study did not use other risk scoring tools like NEWS.

## Conclusions

We found a significant prevalence of *Staphylococcus aureus*, *Pseudomonas aerugenosa,* and *Klebsiella pneumoniae* in COVID-19-associated infections. The presence of raised leukocyte counts and serum lactate should alarm clinicians about the possible presence of infection. There is no significant difference in the presentation of sepsis in the COVID pandemic setting, and among the sepsis accessing tools, SIRS was found out to be a better tool compared to qSOFA and serum lactate. Since this was a single-center study, further research is required on a larger sample size and multicentric to make the results generalized.
